# Characterization of Systemic and Regional Hemodynamics and Vascular Dysfunction in Mice with Fecal Induced Peritonitis

**DOI:** 10.3390/biomedicines10020470

**Published:** 2022-02-18

**Authors:** Forough Jahandideh, Sareh Panahi, Ronan M. N. Noble, Ferrante S. Gragasin, Rachel G. Khadaroo, Kimberly F. Macala, Stephane L. Bourque

**Affiliations:** 1 Department of Anesthesiology & Pain Medicine, University of Alberta, Edmonton, AB T6G 2G3, Canada; jahandid@ualberta.ca (F.J.); sareh@ualberta.ca (S.P.); gragasin@ualberta.ca (F.S.G.); kmacala@ualberta.ca (K.F.M.); 2 Women and Children’s Health Research Institute, University of Alberta, Edmonton, AB T6G 1C9, Canada; noble1@ualberta.ca; 3 Department of Pediatrics, University of Alberta, Edmonton, AB T6G 2G3, Canada; 4 Department of Critical Care Medicine, University of Alberta, Edmonton, AB T6G 2G3, Canada; khadaroo@ualberta.ca; 5 Department of Surgery, University of Alberta, Edmonton, AB T6G 2G3, Canada; 6 Department of Pharmacology, University of Alberta, Edmonton, AB T6G 2G3, Canada

**Keywords:** fecal slurry, hemodynamics, integrated hemodynamics, regional blood flow, sepsis, vascular function

## Abstract

Sepsis is associated with circulatory dysfunction contributing to disturbed blood flow and organ injury. Decreased organ perfusion in sepsis is attributed, in part, to the loss of vasoregulatory mechanisms. Identifying which vascular beds are most susceptible to dysfunction is important for monitoring the recovery of organ function and guiding interventions. This study aimed to investigate the development of vascular dysfunction as sepsis progressed to septic shock. Anesthetized C57Bl/6 mice were instrumented with a fiberoptic pressure sensor in the carotid artery for blood pressure measurements. In subgroups of mice, regional blood flow measurements were taken by positioning a perivascular flow probe around either the left carotid, left renal, or superior mesenteric arteries. Hemodynamic parameters and their responsiveness to bolus doses of vasoactive drugs were recorded prior to and continuously after injection of fecal slurry (1.3 mg/g body weight) for 4 h. Fecal slurry-induced peritonitis reduced mean arterial pressure (62.7 ± 2.4 mmHg vs. 37.5 ± 3.2 mmHg in vehicle and septic mice, respectively), impaired cardiac function, and eventually reduced organ blood flow (71.9%, 66.8%, and 65.1% in the superior mesenteric, renal, and carotid arteries, respectively). The mesenteric vasculature exhibited dysregulation before the renal and carotid arteries, and this underlying dysfunction preceded the blood pressure decline and impaired organ blood flow.

## 1. Introduction

Sepsis is a life-threatening organ dysfunction caused by a dysregulated host response to infection. It is characterized by perturbations in immune, humoral, and circulatory function, which result in increased mortality, and thus needs urgent care, compared with ‘straightforward infections’ [1]. A recent publication based on the Global Burden of Disease, Injuries, and Risk Factors Study estimated that sepsis accounts for almost 20% of all global deaths worldwide [2]. Clinical management of patients with sepsis relies on early recognition of the syndrome, allowing for the rapid institution of antibiotics, source control, and resuscitation with fluids. Vasoactive drugs also constitute a mainstay of treatment for life-threatening hypotension [1]. While treating hypotension is important for survival, the administration of vasopressors, particularly in excess, can compound blood flow disruptions to organs in sepsis, and the resultant organ hypoperfusion may offset the survival benefits of normotension. 

A challenge in studying circulatory changes in sepsis is that vascular dysfunction may be localized to certain vascular beds, contributing to focal regions of impaired blood flow, whereas other regions remain functionally intact [3]. Indeed, compared with global hemodynamic variables, microcirculatory alterations are a stronger predictor of survival outcomes in septic patients [4], underscoring the importance of microvascular regulation and regional control of blood flow in sepsis. Blood vessels rely on neurogenic, metabolic, humoral, and paracrine mediators to control vascular tone and regulate local blood flow. The pathophysiology of vascular dysfunction in sepsis is complex, and the mechanisms dictating blood flow to the respective organs may be such that certain tissues are more vulnerable to blood flow disruptions before others. Although alterations in organ blood flow and, presumably, vascular dysfunction in sepsis, have been reported in the literature [5,6], a fundamental knowledge gap related to the inherent differences in susceptibility to vascular dysfunction among vascular beds and the timeline by which this occurs in sepsis still exists.

Here, we characterized the systemic hemodynamics and regional blood flow changes that occur as sepsis progresses to septic shock. We simultaneously measured systemic blood pressure (BP) as well as blood flow to the brain, kidneys, and mesentery prior to and following induction of sepsis. We further evaluated hemodynamic alterations in response to exogenous pharmacological agents to measure in vivo vascular function in real time. We hypothesized that hemodynamics changes in response to vasoactive agents will decline with the progression of sepsis and the drop in drug-induced alterations in regional blood flow will precede the changes in systemic hemodynamics.

## 2. Materials and Methods

### 2.1. Animals and Treatments

The experimental protocols described herein conform to the guidelines of the Canadian Council on Animal Care, as well as the NIH Guide for the Care and Use of Laboratory Animals, and were approved by the University of Alberta Animal Care and Use Committee. C57Bl/6 male mice (10–12 weeks old) were purchased from Charles River Laboratories Inc. (Saint-Constant, QC, Canada) and group-housed in the Animal Care Facility at the University of Alberta. Environmentally, a 12:12 h light–dark cycle (06:00–18:00) at an ambient temperature of 23 °C with 40–60% relative humidity was maintained, and mice had *ad libitum* access to food and water.

### 2.2. Systemic and Regional Hemodynamic Assessments 

After 1 week of acclimation, mice were anesthetized with inhaled isoflurane (2.5% induction, 1.5% maintenance in pure O_2_) and kept spontaneously breathing via a nose cone on a heated surgical platform connected to a circulating water bath. A rectal thermometer was used to monitor body temperature. Mice were then instrumented with a fiberoptic pressure sensor (FISO-LS-PT9, FISO Technologies Inc., Quebec, QC, Canada) in the right carotid artery for continuous BP and heart rate (HR) measurements. Micro-renathane catheters (0.25 mm OD; Braintree Scientific, Braintree MA) fused to polyethylene tubing (Intramedic, Becton Dickson, I.D. 0.28 mm × O.D. 0.61 mm) were inserted into the left and right femoral veins for fluid and drug delivery, respectively. In subgroups of mice, regional blood flow measurements were made simultaneously with systemic hemodynamics (blood flow was assessed in one single region in each mouse). For these mice, a laparotomy was performed, and a perivascular flow probe (calibrated 0.5 PSL Nano-Prob, Transonic Systems Inc., Ithaca, NY, USA) was positioned around either the left renal or SMA; the abdomen of these mice was then re-sutured. In a separate group of mice, a perivascular flow probe was placed around the left common carotid artery, and the incision was then sutured closed. After instrumentation, mice were given 30 min to stabilize, after which systemic and regional hemodynamics were recorded continuously using PowerLab and LabChart Pro8 software (ADInstruments, Colorado Springs, CO, USA). Baseline hemodynamic parameters were recorded for 15 min prior to administration of a fecal slurry (FS) solution (see below) and continuously thereafter for up to 4 h. For systemic hemodynamics and blood flow patterns, representative waveform segments within each 60 s interval were analyzed and used as a discrete data point; 30 such discrete consecutive time points were then averaged to reflect each 30-min time interval. To analyze hemodynamic responses to vasoactive agents, the maximum change in BP, HR, or organ blood flow that occurred in the 30 s window following injection was quantified; this time-course captured the maximal changes prior to observing compensatory reflexes (e.g., HR changes due to baroreceptor reflexes). The regional vascular resistance index was calculated as a quotient of ΔBP/Δblood flow in that region. Mice were euthanized by exsanguination via excision of the heart under inhaled isoflurane anesthesia at the end of the experiment.

### 2.3. Fecal Slurry Preparation and Induction of Polymicrobial Sepsis in Mice

FS was prepared by collecting the cecal contents of untreated euthanized mice. Cecal contents were suspended in a 5% dextrose solution and then filtered through a 100 µm nylon mesh (to remove large particles). Filtered suspensions were adjusted to yield a final concentration of 80 mg/mL. FS samples were aliquoted and frozen in liquid nitrogen and stored at −80 °C; each aliquot only underwent a single freeze–thaw cycle and was then discarded. For this study, all FS aliquots were taken from a single batch of FS. To induce polymicrobial sepsis, FS was injected at a dose of 1.3 mg/g body weight (BW) IP. Control mice received the equivalent volume of saline. 

### 2.4. Hemodynamic Effects of Vasoactive Agents

**Scheme 1:**
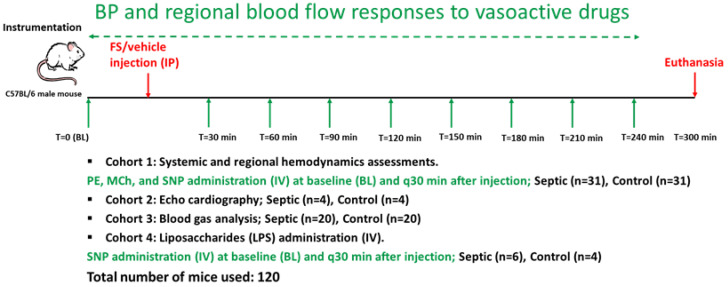
Experimental timeline and the number of mice used for different cohorts of the study. Changes in hemodynamic responses to bolus doses of vasoactive drugs were monitored in vehicle- and FS-treated mice over time for up to 4 h. BP: blood pressure; FS: fecal slurry; IV: intravenous; MCh: methylcholine; PE: phenylephrine; SNP: sodium nitroprusside.

To assess vascular function in vivo with the progression of sepsis, we administered repeat bolus doses of vasoactive drugs (vasoconstrictors and vasodilators) that cause measurable but transient changes in BP. Changes in hemodynamic responses over time were interpreted as alterations in vascular responsiveness to these agents, indicative of changes in vascular function. Briefly, in a subgroup of instrumented mice (as described above), systemic and regional hemodynamic responses to bolus intravenous injections of a single dose of phenylephrine (PE, 10 µg/kg BW), methylcholine (MCh, 1 µg/kg BW), and sodium nitroprusside (SNP, 5 µg/kg BW) were repeatedly assessed. Vasoactive agents were administered prior to injection of the FS (or vehicle) and every 30 min thereafter for up to 4 h. Doses were selected on the basis of our pilot studies in non-septic mice showing that their injection caused pronounced (~15–25 mmHg) but transient (<30 s) changes in BP with minimal effects on HR (<5%). With minimal effects on HR, BP effects could be attributed primarily to changes in vascular tone. The experimental procedure is shown in Scheme 1.

### 2.5. Arterial Blood Gas Analysis

In a separate cohort, otherwise non-instrumented mice were anesthetized with isoflurane (2.5% induction, 1.5% maintenance in pure O_2_) and implanted with a catheter (Micro-renathane, 0.25 mm OD (Braintree Scientific, Braintree, MA, USA) fused to polyethylene tubing (Intramedic, Becton Dickson, Becton, NJ, USA, I.D. 0.28 mm × O.D. 0.61 mm)) in the left carotid artery for blood collection. Because blood gas analysis required sample volumes of ~150 µL, serial assessments were not performed due to the potential impact of repeated sampling on hemodynamics and overall survival. Arterial blood was collected under anesthesia in calcium-balanced heparin-coated syringes (Blood Gas Monovette, Sarstedt Inc., Montreal, QC, Canada) at 0.5 h, 1 h, 2 h, and 4 h post-injection of FS or the vehicle, then immediately analyzed by a blood gas analyzer (AB Flex 80, Radiometer Canada, Mississauga, ON, Canada).

### 2.6. Echocardiography

Cardiac function was assessed by transthoracic echocardiography (Vevo 2100, Visualsonics, Toronto, ON, Canada) using a linear array transducer ranging from 18 to 38 MHz (MS400, Visualsonics, Toronto, ON, Canada). A single operator blinded to the experimental groups performed all echocardiographic measurements. Animals were anesthetized with isoflurane (2.5% induction, 1.5% maintenance in pure O_2_) and then fixed to a heated platform. The chest was depilated and echocardiography was performed under isoflurane anesthesia in the supine position using a heated ultrasound transmission gel. The heart was imaged via two-dimensional B mode in a parasternal short axis view and once a clear view had been established, an M mode cursor was placed perpendicular to the anterior and posterior wall of the left ventricle and parallel to the septum. Left ventricle internal diameters were obtained during the end-diastole and end-systole. Ejection fraction and stroke volume were then calculated from the ventricular volumes computed from M mode images; heart rates were measured in the same cardiac cycles.

### 2.7. Intravenous Administration of Lipopolysaccharide (LPS) to Mice

In a separate cohort of mice (instrumented with a carotid pressure sensor for BP measurements and perivascular flow probes to measure carotid or SMA blood flow, as described above), LPS (7 mg/kg) or an equivalent volume of saline was injected intravenously in the left femoral vein. In these mice, continuous systemic and regional hemodynamics, and hemodynamic responses to repeat administration of single doses of SNP were assessed prior to and for 4 h following administration of LPS. The dose of LPS was chosen in line with pilot studies showing a comparable mortality within 4 h to FS injection (at 1.3 mg/g BW).

### 2.8. Statistical Analysis

Due to the exploratory nature of the work, there were different endpoints assessed throughout the study, including systemic and regional hemodynamics, response to vasoactive drugs as a measure of in vivo vascular function, heart function, and changes in lactate and arterial blood gas parameters. Sample sizes for various endpoints were calculated on the basis of perceived differences and variances from pilot experiments. The confidence level and power were set at 95% (α = 0.05) and 80%, respectively. Data were analyzed by two-way analysis of variance for the effects of FS and time, followed by the Holm–Sidak *post hoc* test for multiple comparisons using Prism 8.0 (GraphPad Software, Inc., San Diego, CA, USA); *p* < 0.05 was considered statistically significant. 

## 3. Results

### 3.1. FIP Changes Systemic Hemodynamics and Heart Function 

**Figure 1:**
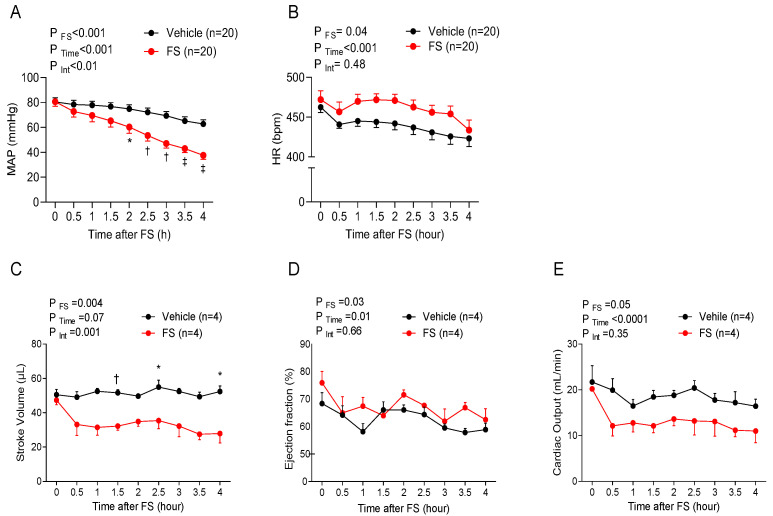
Fecal slurry (FS)-induced peritonitis affected (**A**) mean blood pressure (MAP), (**B**) heart rate (HR), (**C**) stroke volume, (**D**) ejection fraction, and (**E**) cardiac output. Data were analyzed by two-way ANOVA for the effects of FS and time, followed by the Holm–Sidak *post hoc* test for multiple comparisons. * *p* < 0.05, ^†^ *p* < 0.01, and ^‡^ *p* < 0.001 compared with the vehicle at the same time.

FS-induced peritonitis (FIP) caused a progressive decline in mean BP over time that was first evident 2 h after sepsis induction (Figure 1A). This was accompanied by an immediate and sustained increase in HR (Figure 1B). FIP also caused a 32% reduction in stroke volume (*p* = 0.001) within 1.5 h of FS injection (Figure 1C) and a modest increase in ejection fraction (Figure 1D). The overall effect of FIP was a 46% reduction in cardiac output compared with a 20% reduction in the vehicle-treated mice (Figure 1E). The magnitude of this reduction was mitigated compared with stroke volume, possibly due to compensatory increases in HR and the ejection fraction.

### 3.2. FIP Changes Regional Hemodynamics 

**Figure 2:**
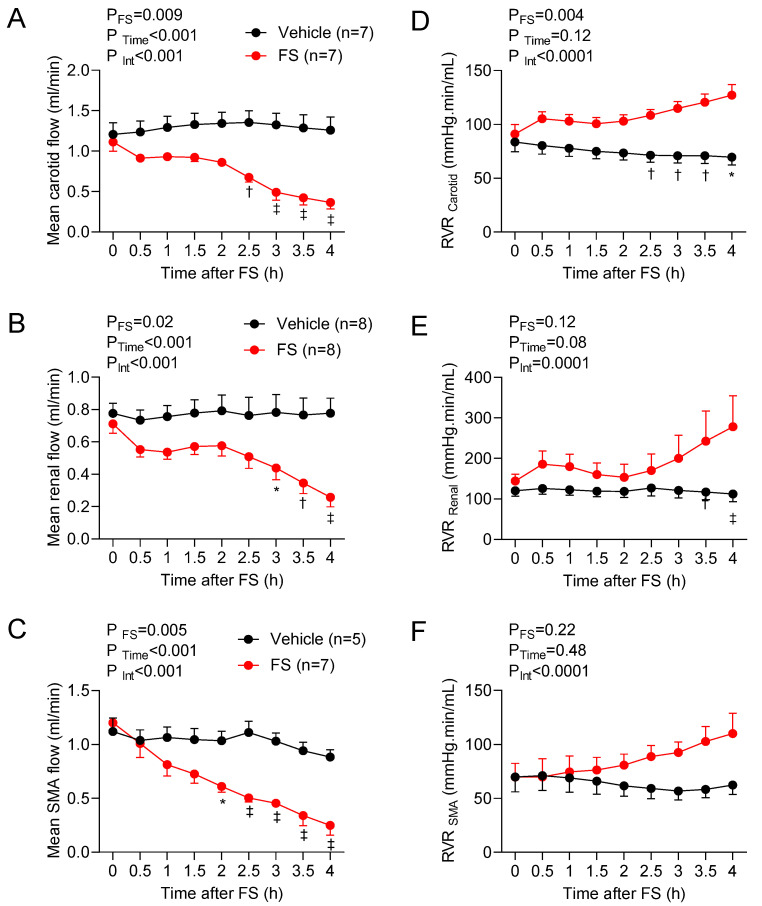
Fecal-slurry (FS)-induced peritonitis caused a progressive decline in mean blood flow through (**A**) the carotid artery, (**B**) the renal artery, and (**C**) the superior mesenteric artery (SMA) and a progressive increase in regional vascular resistance (RVR) of (**D**) the carotid artery, (**E**) the renal artery, and (**F**) the superior mesenteric artery (SMA). Data were analyzed by two-way ANOVA for effects of FS and time, followed by the Holm–Sidak *post hoc* test for multiple comparisons. * *p* < 0.05, ^†^ *p* < 0.01, and ^‡^ *p* < 0.001 compared with the vehicle at the same time.

Evidence of reduced organ blood flow began to manifest over a similar time-course to BP; SMA blood flow was reduced 2 h post-injection of FS (Figure 2C), whereas carotid (Figure 2A) and renal blood flow (Figure 2B) were reduced at 2.5 h and 3 h post-injection of FS, respectively. These changes in blood flow could not be attributed solely to reduced perfusion pressure, as denoted by the progressive increase in the regional vascular resistance profiles in the studied vascular beds (Figure 2D–F). 

### 3.3. FIP Changes Arterial Blood Gas Parameters 

FIP had profound effects on arterial blood gas parameters (Table S1). Oxygen content was primarily affected at the initial phase of sepsis. Conversely, changes in pH and partial pressure of O_2_ were not evident until 4 h after FS injection (*p* = 0.007 and *p* = 0.02, respectively). Hematocrit percentage (*p* = 0.02) and hemoglobin content (*p* = 0.0002) increased in FIP mice compared with the vehicle-treated mice, while the oxygen saturation of hemoglobin reduced (*p* = 0.01). FS injection reduced sodium and chloride levels and increased potassium levels. Standard bicarbonate and standard base excess, which reflects the quantity of acid or base required to restore normal blood pH under standard conditions [7], was reduced in FIP mice. Differences in the base levels were most apparent at t = 2 h and t = 4 h (*p* = 0.05 and *p* = 0.0008, respectively), whereas bicarbonate levels decreased at t = 4 h in FIP mice (*p* = 0.0003). The comparable respiratory component (partial pressure of CO_2_) but different standard base excess values between FS and vehicle-treated mice suggests that the metabolic component of the acid–base balance is perturbed in the former group. Plasma lactate levels tended to elevate (+54%) within 0.5 h of FS injection (*p* = 0.06) and reached the highest levels 4 h after FS injection (+80%; *p* = 0.0006). The persistent increase in plasma lactate in combination with reduced blood pH suggested lactic acidosis and a shift towards anaerobic respiration—the most common cause of metabolic acidosis [8]—in septic mice.

### 3.4. FIP Changes Systemic Hemodynamic Responses to Vasoactive Drugs

**Figure 3:**
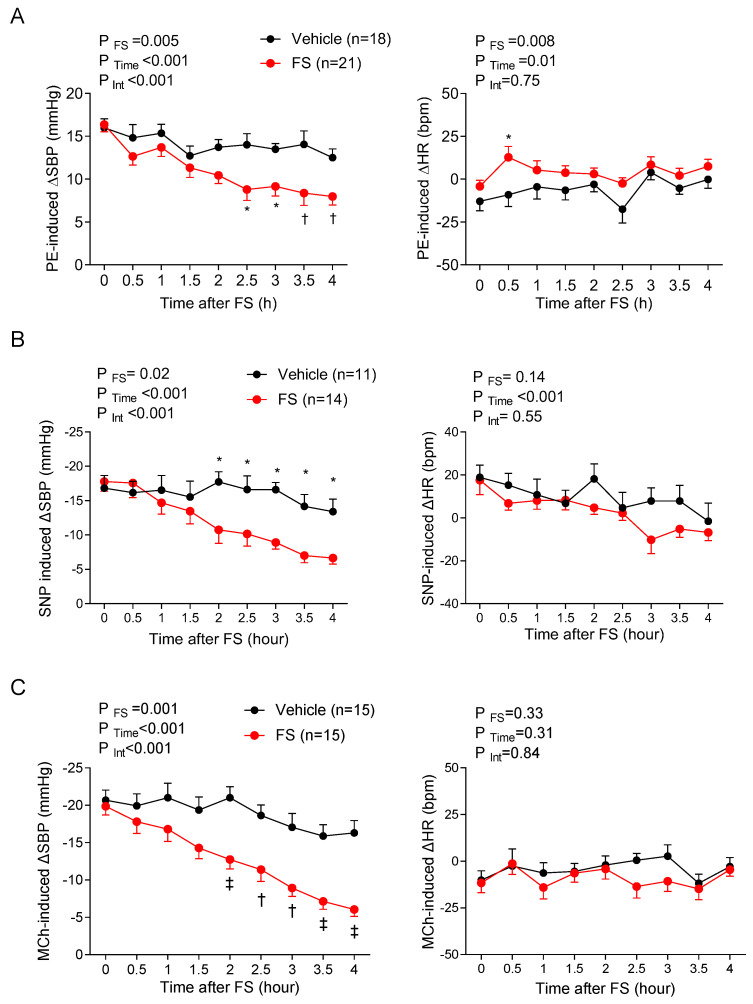
Fecal slurry (FS)-induced peritonitis caused a progressive reduction in systolic blood pressure (SBP) response (left panels) to bolus doses of (**A**) phenylephrine (PE), (**B**) sodium nitroprusside (SNP), and (**C**) methylcholine (MCh) over time in mice, with minimal changes in heart rate (HR, right panels). Data were analyzed by two-way ANOVA for the effects of FS and time, followed by the Holm–Sidak *post hoc* test for multiple comparisons. * *p* < 0.05, ^†^ *p* < 0.01, and ^‡^ *p* < 0.001 compared with the vehicle at the same time.

Injection of PE caused a transient rise in BP (Figure 3A, left panel) with minimal changes in HR (Figure 3A, right panel), reflecting a systemic vasoconstrictor response in mice prior to FS injection. FIP caused a gradual decline in BP responses to PE over time (*p* < 0.01), first evident at t = 2.5 h after FS injection (*p* = 0.01), and culminated in a >50% loss of responsiveness by 4 h post-injection of FS. Administration of bolus doses of SNP (Figure 3B) and MCh (Figure 3C) caused transient decreases in BP that were similar in both groups prior to FS injection. FIP caused the BP responses to SNP and MCh to gradually diminish over time; changes were evident from t = 2 h post FS injection (*p* < 0.001) onwards. By the end of the experiment (t = 4 h), BP responses to MCh and SNP declined by approximately 70% in FIP mice compared with vehicle-treated mice (*p* < 0.001).

### 3.5. FIP Changes Regional Hemodynamic Responses to Vasoactive Drugs

**Figure 4:**
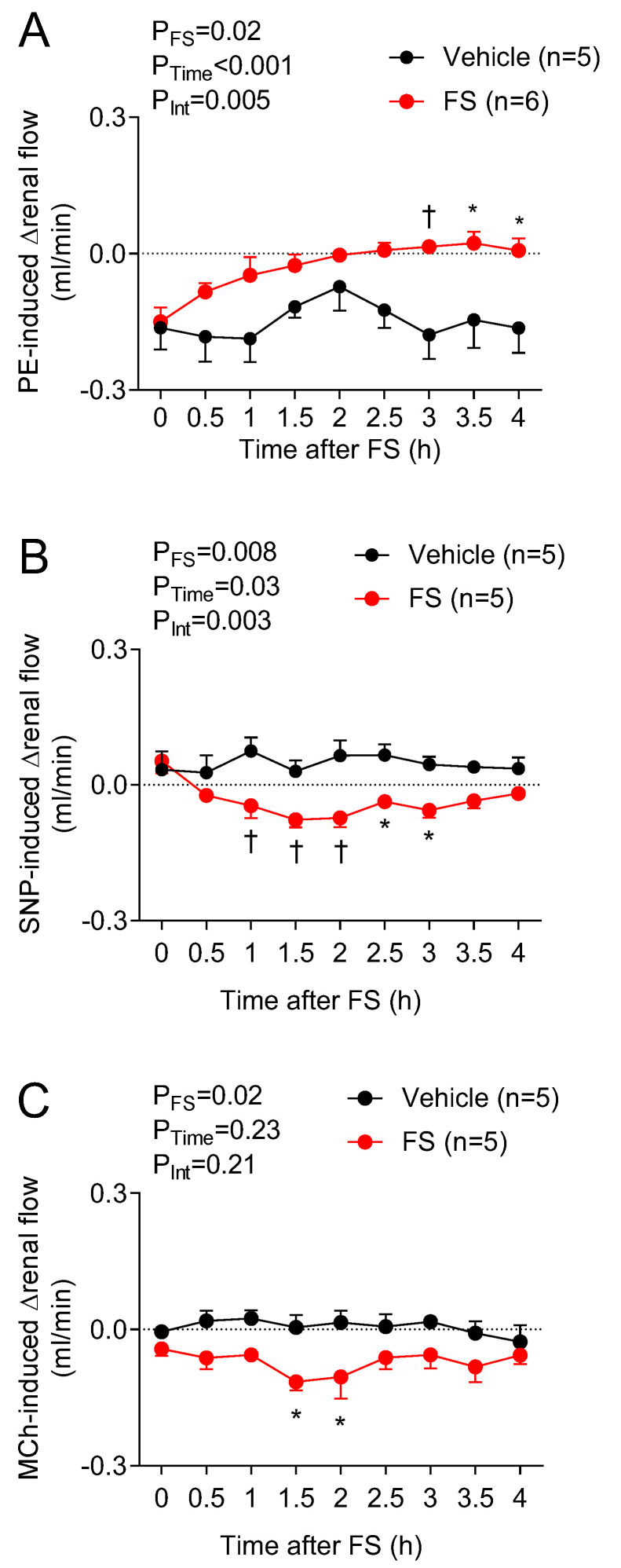
Fecal slurry (FS)-induced peritonitis caused progressive changes in renal blood flow responses to repeat administration of bolus doses of (**A**) phenylephrine (PE), (**B**) sodium nitroprusside (SNP), and (**C**) methylcholine (MCh) over time in mice. Data were analyzed by two-way ANOVA for the effects of FS and time, followed by the Holm–Sidak *post hoc* test for multiple comparisons. * *p* < 0.05 and ^†^ *p* < 0.01 compared with the vehicle at the same time.

In both control and FS-injected groups, vasoactive drugs (Figure S1A–C) caused only modest changes in carotid blood flow, despite the pronounced effects on BP. There was no effect of FIP on carotid blood flow patterns. In control mice, PE reduced renal blood flow (Figure 4A), whereas SNP (Figure 4B) and MCh (Figure 4C) had little overall effect. In FIP mice, renal blood flow responses to PE gradually declined over time, with the differences being most apparent after 3 h of FS injection (Figure 4A). SNP (Figure 4B) and MCh (Figure 4C) caused reduced blood flow in FIP mice, with the differences being most apparent between 1 and 3 h post-injection of FS.

**Figure 5:**
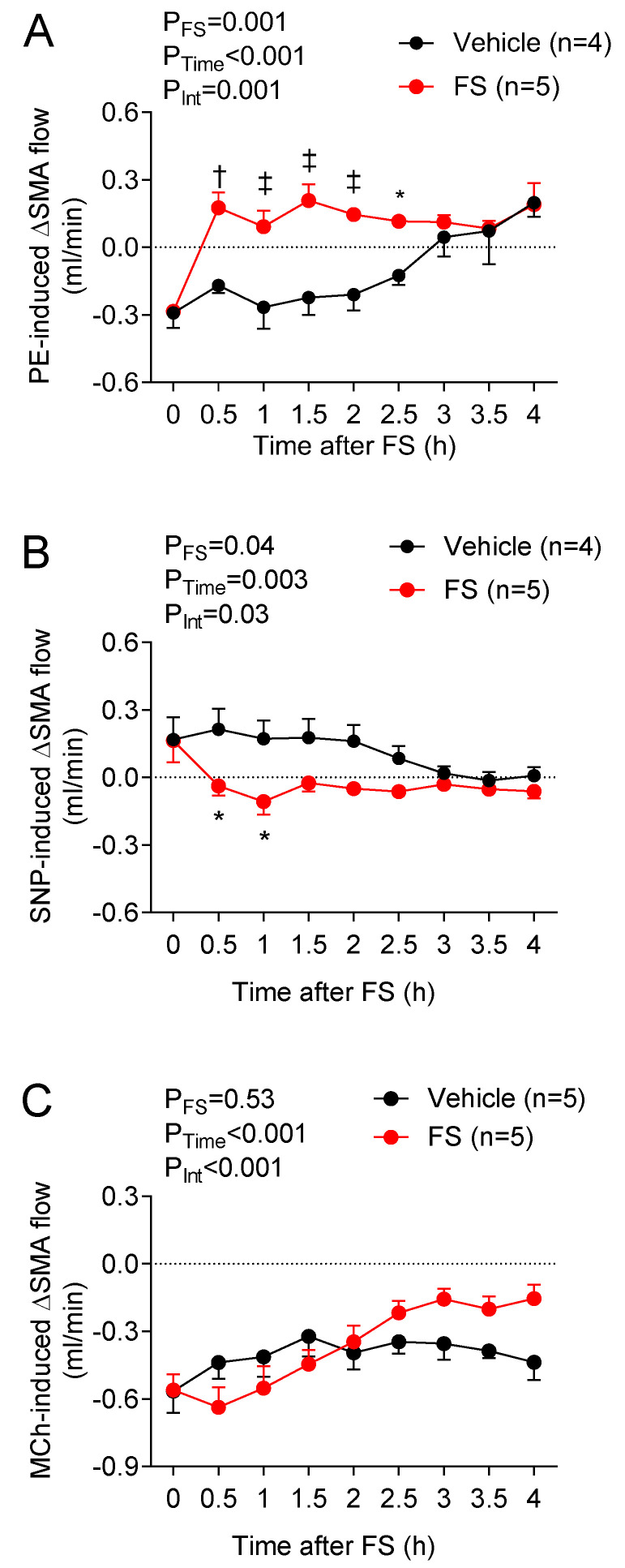
Fecal slurry (FS)-induced peritonitis caused progressive changes in superior mesenteric artery (SMA) blood flow responses to bolus doses of (**A**) phenylephrine (PE), (**B**) sodium nitroprusside (SNP), and (**C**) methylcholine (MCh) over time in mice. Data were analyzed by two-way ANOVA for the effects of FS and time, followed by the Holm–Sidak *post hoc* test for multiple comparisons. * *p* < 0.05, ^†^ *p* < 0.01, and ^‡^ *p* < 0.001 compared with the vehicle at the same time.

In contrast to the carotid and renal vascular beds, all vasoactive agents caused changes in SMA blood flow. In control mice, PE reduced SMA blood flow, although these effects waned over time (Figure 5A). Nevertheless, FIP mice exhibited a marked reversal of response within 0.5 h of FS administration, suggesting an accelerated impairment of PE responsiveness. Similar but opposite effects were observed with SNP, such that SMA blood flow in control mice initially increased, but the responses gradually declined over time (Figure 5B), whereas FIP showed an accelerated loss of responsiveness that was evident within 0.5 h of FS injection. MCh reduced SMA blood flow in both control and FIP groups, and this effect gradually declined over time in FIP mice only (Figure 5C). 

### 3.6. SNP-Induced Changes in Hemodynamics in LPS-Treated Mice

In a separated cohort of mice, we tested whether proximity to the source of infection (FS in this study) contributed to the observed effects of sepsis on SMA blood flow dysregulation. Similar to the FIP model, LPS injection caused a gradual decline in BP responsiveness (Figure S2A), with modest effects on HR (Figure S2B). As observed with FIP, LPS injection had little effect on carotid blood flow responsiveness to SNP (Figure S2C) but caused pronounced changes in SMA blood flow responsiveness to this drug (Figure S2D). This was characterized by a reversal of SMA blood flow responses that were evident 1 h post-injection of endotoxin (Figure S2D).

## 4. Discussion

The objective of this study was to evaluate the intrinsic differences in susceptibility to dysfunction among vascular beds in sepsis, as well as the timeline at which the vascular dysfunction occurs. Using a murine model of FIP, we showed that the development of vascular dysfunction and regional blood flow perturbation in sepsis is not uniform; instead, certain vascular beds (notably the mesenteric vasculature) exhibit dysregulation before the others, and this underlying dysfunction precedes the BP decline and impaired blood flow to these organs. 

The FIP model of polymicrobial sepsis is commonly used and generally well characterized, though the degree of peritonitis induced in the present study was comparatively severe, causing circulatory collapse within 6 h of FS injection. FIP altered cardiovascular function, as observed by increasing HR and diminishing BP over time. The former may constitute an adaptive response to mitigate the fall in cardiac output [9,10]. However, since the cardiac changes did not temporally coincide with the fall in BP, it is likely that a progressive loss of systemic vascular resistance was the primary factor associated with circulatory compromise herein [11]. 

The gradual decline in BP was accompanied by impaired regional blood flow patterns. While we observed differences among regions, these were less pronounced than expected, given that cerebral and renal blood flow are autoregulated, whereas blood flow to the intestine is less so [12,13,14]. Indeed, the linear decline in blood flow in the mesenteric circulation mirrored the gradual decline in BP, consistent with a progressive decline in perfusion pressure. In contrast, carotid and renal blood flow in FIP mice exhibited a biphasic pattern, characterized by an initial plateau phase (reflecting autoregulation), followed by a progressive decline thereafter (wherein the fall in perfusion pressure exceeded the autoregulatory range) [15]. It should be noted that while the majority of studies have reported reduced renal blood flow in experimental models of sepsis, others have reported unchanged or even increased blood flow to the kidneys [16]. This apparent discrepancy may be reconciled by the fact that cardiac output is a primary determinant of blood flow patterns to the kidneys [16]. Reduced cardiac output, as seen in the present study, is often accompanied by decreased renal blood flow, while preserved or high cardiac output predicted unchanged or increased renal blood flow in experimental models of sepsis [16]. As differences in wet and dry tissue weights were comparable between the two groups (data not shown), increased vascular permeability and tissue edema are less likely to be attributed to the increased vascular resistance and decreased BP, consistent with other models of sepsis [17]. 

The impaired organ blood flow was associated with a number of systemic metabolic changes, including an increase in plasma lactate levels with concurrent metabolic acidosis [8], which is associated with increased mortality in both emergency departments and hospitalized patients [18]. Though elevated lactate levels could also reflect respiratory compromise, the increased blood oxygen content suggested that the increased lactate levels were primarily due to the reduced oxygen uptake and/or release rather than oxygen transport [7]. While the observed increase in oxygen content in FIP mice was due to hemoconcentration, the reduced partial pressure of O_2_ and the increased lactate levels, in conjunction with impaired organ blood flow in FIP mice, indicate the development of ischemic hypoxia in septic mice. 

The gradual decline in regional blood flow occurring after 1.5 h was greater in magnitude than the drop in perfusion pressure (as reflected by the increased regional vascular resistance) without pronounced tissue edema, suggesting a progressive decline in the vascular control mechanisms that maintain tonic vasodilation. To gain additional insights into how the vascular mechanisms regulating organ blood flow were affected in sepsis, we assessed hemodynamic responses to several vasoactive agents—an approach that integrated all regulatory systems at play within a particular vascular bed, including those regulating the vascular tone of resistance arterioles. Thus, the blood flow through that vascular bed is influenced by all the homeostatic mechanisms at play in all downstream vessels. To the best of our knowledge, the present study was the first to interrogate the mechanisms of vascular dysfunction by using in vivo methods to serially assess vascular function over time by administering vasoactive agents. The doses of vasoactive agents used caused minimal changes in HR; therefore, the BP effects could be attributed predominantly to changes in vascular resistance. In non-septic control mice, PE caused a transient rise in BP, reflecting increased systemic vasoconstriction, whereas SNP and MCh resulted in hypotensive responses, reflecting a systemic vasodilatory effect. In FIP mice, BP responses to tested vasoactive agents began to diminish 2–2.5 h post-injection of FS, suggesting a time-dependent loss of vascular reactivity (i.e., vasoplegia), but loss of responsiveness was similar with each pharmacological agent. In contrast, the analysis of regional blood flow patterns revealed marked differences. In control mice, none of the agents had marked effects on carotid or renal blood flow, except for PE, reflecting intact autoregulatory systems. Interestingly, direct pharmacological agonism with PE, SNP, and MCh has been shown to affect blood flow in these beds [19,20,21,22], suggesting that the direct vascular effects of these agonists interfere with autoregulatory mechanisms in these vascular beds. That no such changes were observed in non-septic control mice may be related to the low doses of agents used in the present study. In contrast, the superimposable blood flow profiles between the control and FIP mice with all administered agents suggests that carotid blood flow is less prone to perturbations when vascular control systems go awry in sepsis. Though reduced responsiveness to all agents was observed in the renal artery, these differences did not manifest as quickly as in the SMA, again possibly due to compensation by autoregulatory mechanisms. In the mesenteric bed, reduced and increased blood flow with PE and SNP, respectively, can be attributed to direct actions on vascular resistance, rather than their effects on perfusion pressure. Although the effects of PE and SNP waned over time even in non-septic mice, likely reflecting the well described phenomena of tachyphylaxis with these agents [23,24], the time-course in septic animals was far more rapid. In septic mice, SMA blood flow changes in response to PE and SNP were disturbed within 0.5 h post-injection of FS, suggesting that sepsis causes a marked acceleration in vascular unresponsiveness to these drugs. The rapid onset of vasoplegia in the SMA could not be attributed to the proximity of the intraperitoneal FS injection, as a similar time-course of dysfunction was observed in mice systemically injected with LPS. Interestingly, the observed SMA blood flow dysregulation in response to vasoactive drugs was concurrent with the immediate rise in plasma lactate levels. This shows that metabolic and vascular derangements occur early on in sepsis before changes in systemic hemodynamics are apparent. Therefore, regional hemodynamic assessment is critical in determining the status of vascular integrity for the timely institution of potential treatments before permanent vasculature damage and organ dysfunction occur. These novel findings are important for devising intervention strategies to specifically promote blood flow in dysfunctional regions while preventing life-threatening hypotension.

SNP stimulates soluble guanyl cyclase (sGC) in smooth muscle cells, catalyzing the dephosphorylation of guanosine triphosphate to cyclic guanosine monophosphate, which induces smooth muscle relaxation through several mechanisms, including sequestering calcium and activation of cell membrane potassium channels [25,26]. In conditions of oxidative stress, oxidation of the ferrous heme in sGC generates heme-free sGC [27], which is susceptible to ubiquitination and subsequent breakdown [28]. Although heme-free sGC has preserved basal activity, it cannot be activated by nitric oxide (NO) [29]. The reversal of blood flow in the mesentery within 0.5 h of FIP suggests that a disturbed sGC–cyclic guanosine monophosphate signaling pathway is evident in this vascular bed as a result of FIP in mice earlier than in other vascular beds.

PE, the α1 selective adrenergic receptor agonist, causes vasoconstriction and increases systemic vascular resistance and arterial blood pressure. Vascular hyporesponsiveness to vasopressors has been characterized in septic models [30]. Dysfunction, desensitization and reduction of α receptors; inactivation of catecholamines by oxidation, and adrenal insufficiency are among the suggested mechanisms for vascular hyporesponsiveness to vasopressors [31,32,33]. A reduction in the number of hepatic α1 adrenergic receptors has been reported in a rat model of sepsis [34]. The hypothalamic–pituitary–adrenal axis, the main neuroendocrine structure regulating the adaptive response to different stressors, is responsible for catecholamine secretion, vasopressin release, and cytokine activation in sepsis. Hypothalamic–pituitary–adrenal axis dysfunction results in reduced production of the corticotropin-releasing hormone, adrenocorticotropic hormone, and cortisol, or causes relative hormonal deficiency by affecting their receptors [35,36]. The immediate effect of FIP on reversing SMA blood flow response to PE may suggest that mesenteric α1 receptors are affected earlier than these receptors in other vascular beds following FIP in mice. 

Interestingly, MCh caused a pronounced lowering of SMA blood flow, suggesting that its effect of reducing perfusion pressure predominates over its direct vasodilatory effects in the mesenteric circulation [37]. By extension, this suggests that the BP-lowering effect of MCh may be mediated by vasodilation of blood vessels supplying the skeletal muscle, which muscarinic receptors are known to populate [38], rather than directly affecting the mesenteric circulation. With the progression of sepsis, changes in the responsiveness to MCh were relatively slower compared with those to SNP, despite the reliance on NO–sGC signaling [37]. This discrepancy may be reconciled by the fact that MCh can elicit vasodilation via multiple mechanisms (e.g., NO, EDH, etc.); in pathological conditions, alternative mechanisms may compensate for the loss of vascular regulatory systems. 

Although we have identified that NO signaling and sympathetic stimulation-dependent pathways are altered early regionally in sepsis, detailed studies are required to understand the complex mechanisms governing the susceptibility of certain vascular beds to dysfunction over others. Blood flow responses to administered agents, as performed herein, combine the integrated effects of direct vascular effects, changes in perfusion pressure (caused by the vasoactive effects of agents in other vascular beds), and autoregulatory responses. Due to the systemic nature of sepsis and the breadth of metabolic, circulatory, and immune perturbations characterizing its progression, it is likely that multiple mechanisms are involved. For example, in addition to the disturbed sGC–cyclic guanosine monophosphate signaling pathway [39], receptor downregulation (due to internalization and decreased expression) is another mechanism underling the development of vasoplegia in sepsis [32,33]. Additionally, changes in metabolic function have been reported to directly affect vascular function. Lactic acidosis initiates intracellular signaling cascades in both endothelial and vascular smooth muscle cells, resulting in alterations in the calcium transient and the number of adrenoreceptors on the cell surface [30,40], the opening of ATP-sensitive potassium channels [41], and an increase in the expression of inducible nitric oxide synthase in the endothelium and vascular smooth muscle cells [42,43]. In addition to the well-studied effects of metabolic stresses on endothelial cells (and vascular function), emerging data suggest that the vascular endothelium may play a pivotal role in metabolic homeostasis regulation and that endothelial dysfunction directly contributes to the development of metabolic disorders [44]. The importance of uninterrupted energy production in sepsis has been reported recently [45]. Thus, the combined effects that result in perturbed vascular signaling and dysregulated energy homeostasis are likely to be multifaceted in sepsis and require further interrogation. 

Regardless of the mechanism, vascular dysfunction and vasoplegia are localized to certain regions. We have shown that inherent differences exist between different vascular beds and, surprisingly, the dysfunction in certain beds (mesenteric) progresses more quickly compared with other vascular beds. Moreover, our data suggest that the development of regional vascular dysfunction could be underway without evidence of BP dysregulation or a change in total organ blood flow. Selective targeting of damaged vascular beds to improve regional blood flow [46] is a potential novel treatment which can eventually prevent or at least delay the onset of multi-organ damage and death in sepsis.

Notable limitations of our study include the lack of an antibiotic treatment in this relatively severe model of sepsis. Antibiotics were withheld because the time period studied herein reflects what could be considered the acute (or hyper-acute) phase of sepsis, in which patients would not typically be treated; in this way, the unabated progression of sepsis to septic shock could be studied. Nevertheless, since antibiotics constitute a mainstay treatment of sepsis, their impact on sepsis progression (and the hemodynamic consequences thereof) remains an important topic. Another limitation was the use of saline as a vehicle, rather than a 5% dextrose solution (in which the FS was prepared). Although our pilot studies revealed that dextrose administration had a minor influence on blood glucose levels in non-septic rats, the use of saline in control mice nonetheless confounds the interpretation of altered glucose handling in FS-treated mice, and follow-up studies are needed to address this point. A final limitation of the study is the use of anesthetic throughout. Although isoflurane is an agent of choice due to its modest cardiovascular, respiratory, and immunomodulatory effects in otherwise healthy individuals [47,48], in high-risk patients with pre-existing immune dysfunction or multiple organ failure, the choice/influence of anesthetics on the inflammatory response may have clinical implications [49]. Therefore, the results presented herein must be interpreted with caution. Notwithstanding, it is noteworthy that ICU patients are often exposed to anesthetic agents for prolonged periods; therefore, the complex side effects of anesthetics may be clinically important in this population.

## 5. Conclusions

In summary, the data presented herein suggest that vascular dysfunction occurs prior to the manifestation of changes in systemic hemodynamics as sepsis progresses over time. Local perturbations in organ blood flow are potentially due to the greater susceptibility of certain vascular beds (i.e., mesenteric arteries) to dysfunction than others. Therefore, regional hemodynamic assessment is critical in determining the status of vascular integrity for timely institution of potential treatments to prevent permanent vasculature damage and organ dysfunction development.

## Data Availability

Additional data are available online as supplementary data. The raw data and experimental materials used in this study are available upon reasonable request to the corresponding author.

## Supplementary Materials

The following supporting information can be downloaded at: https://www.mdpi.com/article/10.3390/biomedicines10020470/s1. Figure S1: Fecal slurry-induced peritonitis (FIP) had little effect on carotid blood flow responses to repeat administration of bolus doses of (A) PE, (B) SNP, and (C) MCh in mice. Data were analyzed by two-way analysis of variance (ANOVA) for the effects of FS and time. Figure S2. Lipopolysaccharide (LPS) administration caused progressive reduction in systolic blood pressure response (A), no changes in heart rate (B) and carotid flow (C), and a substantial reduction in the response of SMA flow to bolus doses of SNP (D) in mice. Data were analyzed by two-way analysis of variance (ANOVA) for the effects of FS and time, followed by the Holm–Sidak *post hoc* test for multiple comparisons. * *p* < 0.05 and ^‡^ *p* < 0.001 compared with the vehicle at the same time. Table S1. Biochemical parameters of blood gas analysis in fecal slurry (FS)-injected and vehicle groups.

## References

[1] Singer M., Deutschman C.S., Seymour C.W., Shankar-Hari M., Annane D., Bauer M., Bellomo R., Bernard G.R., Chiche J.-D., Angus D.C. (2016). The Third International Consensus Definitions for Sepsis and Septic Shock (Sepsis-3). JAMA.

[2] Rudd K.E., Johnson S.C., Agesa K.M., Shackelford K.A., Tsoi D., Kievlan D.R., Colombara D.V., Ikuta K.S., Kissoon N., Finfer S. (2020). Global, regional, and national sepsis incidence and mortality, 1990–2017: Analysis for the Global Burden of Disease Study. Lancet.

[3] Sharawy N. (2014). Vasoplegia in septic shock: Do we really fight the right enemy?. J. Crit. Care.

[4] De Backer D., Donadello K., Sakr Y., Ospina-Tascon G., Salgado D., Scolletta S., Vincent J.L. (2013). Microcirculatory alterations in patients with severe sepsis: Impact of time of assessment and relationship with outcome. Crit. Care Med..

[5] Brenner M., Schaer G.L., Mallory D.L., Suffredini A.F., Parrillo J.E. (1990). Detection of renal blood flow abnormalities in septic and critically ill patients using a newly designed indwelling thermodilution renal vein catheter. Chest.

[6] De Backer D., Creteur J., Preiser J.C., Dubois M.J., Vincent J.L. (2002). Microvascular blood flow is altered in patients with sepsis. Am. J. Resp. Crit. Care.

[7] Seeger C., Higgins C. (2014). Acute Care Testing Handbook.

[8] Casaletto J.J. (2005). Differential diagnosis of metabolic acidosis. Emerg. Med. Clin. N. Am..

[9] Merx M.W., Weber C. (2007). Sepsis and the heart. Circulation.

[10] Flynn A., Chokkalingam Mani B., Mather P.J. (2010). Sepsis-induced cardiomyopathy: A review of pathophysiologic mechanisms. Heart Fail. Rev..

[11] Beale R.J., Hollenberg S.M., Vincent J.L., Parrillo J.E. (2004). Vasopressor and inotropic support in septic shock: An evidence-based review. Crit. Care Med..

[12] Bevan J.A., Hwa J.J. (1985). Myogenic tone and cerebral vascular autoregulation: The role of a stretch-dependent mechanism. Ann. Biomed. Eng..

[13] Navar L.G. (1978). Renal autoregulation: Perspectives from whole kidney and single nephron studies. Am. J. Physiol..

[14] Carlson B.E., Arciero J.C., Secomb T.W. (2008). Theoretical model of blood flow autoregulation: Roles of myogenic, shear-dependent, and metabolic responses. Am. J. Physiol. Heart Circ. Physiol..

[15] Klabunde R.E. (2012). Cardiovascular Physiology Concepts.

[16] Langenberg C., Bellomo R., May C., Wan L., Egi M., Morgera S. (2005). Renal blood flow in sepsis. Crit. Care.

[17] Piper R.D., Pitt-Hyde M., Li F., Sibbald W.J., Potter R.F. (1996). Microcirculatory changes in rat skeletal muscle in sepsis. Am. J. Respir Crit. Care Med..

[18] Jansen T.C., van Bommel J., Bakker J. (2009). Blood lactate monitoring in critically ill patients: A systematic health technology assessment. Crit. Care Med..

[19] McGiff J.C., Burns R.B., Blumenthal M.R. (1967). Role of acetylcholine in the renal vasoconstrictor response to sympathetic nerve stimulation in the dog. Circ. Res..

[20] Ito S., Carretero O.A., Abe K. (1995). Nitric oxide in the regulation of renal blood flow. New Horiz..

[21] Toda N., Ayajiki K., Okamura T. (2009). Cerebral Blood Flow Regulation by Nitric Oxide: Recent Advances. Pharmacol. Rev..

[22] Scremin O.U., Jenden D.J. (1996). Cholinergic control of cerebral blood flow in stroke, trauma and aging. Life Sci..

[23] Amaranath L., Kellermeyer W.F. (1976). Tachyphylaxis to sodium nitroprusside. Anesthesiology.

[24] Dora K.A., Hinton J.M., Walker S.D., Garland C.J. (2000). An indirect influence of phenylephrine on the release of endothelium-derived vasodilators in rat small mesenteric artery. Br. J. Pharmacol..

[25] Hottinger D.G., Beebe D.S., Kozhimannil T., Prielipp R.C., Belani K.G. (2014). Sodium nitroprusside in 2014: A clinical concepts review. J. Anaesthesiol. Clin. Pharmacol..

[26] Charlton M., Sims M., Coats T., Thompson J.P. (2017). The microcirculation and its measurement in sepsis. J. Intensive Care Soc..

[27] Stasch J.P., Schmidt P.M., Nedvetsky P.I., Nedvetskaya T.Y., HS A.K., Meurer S., Deile M., Taye A., Knorr A., Lapp H. (2006). Targeting the heme-oxidized nitric oxide receptor for selective vasodilatation of diseased blood vessels. J. Clin. Investig..

[28] Hoffmann L.S., Schmidt P.M., Keim Y., Schaefer S., Schmidt H.H., Stasch J.P. (2009). Distinct molecular requirements for activation or stabilization of soluble guanylyl cyclase upon haem oxidation-induced degradation. Br. J. Pharmacol..

[29] Roy B., Mo E., Vernon J., Garthwaite J. (2008). Probing the presence of the ligand-binding haem in cellular nitric oxide receptors. Br. J. Pharmacol..

[30] Levy B., Collin S., Sennoun N., Ducrocq N., Kimmoun A., Asfar P., Perez P., Meziani F. (2010). Vascular hyporesponsiveness to vasopressors in septic shock: From bench to bedside. Intensive Care Med..

[31] Macarthur H., Westfall T.C., Riley D.P., Misko T.P., Salvemini D. (2000). Inactivation of catecholamines by superoxide gives new insights on the pathogenesis of septic shock. Proc. Natl. Acad. Sci. USA.

[32] Takakura K., Taniguchi T., Muramatsu I., Takeuchi K., Fukuda S. (2002). Modification of alpha(1)-adrenoceptors by peroxynitrite as a possible mechanism of systemic hypotension in sepsis. Crit. Care Med..

[33] Burgdorff A.M., Bucher M., Schumann J. (2018). Vasoplegia in patients with sepsis and septic shock: Pathways and mechanisms. J. Int. Med. Res..

[34] Mcmillan M., Chernow B., Roth B.L. (1986). Hepatic Alpha-1-Adrenergic Receptor Alteration in a Rat Model of Chronic Sepsis. Circ. Shock.

[35] Prigent H., Maxime V., Annane D. (2004). Clinical review: Corticotherapy in sepsis. Crit. Care.

[36] Gheorghita V., Barbu A.E., Gheorghiu M.L., Caruntu F.A. (2015). Endocrine dysfunction in sepsis: A beneficial or deleterious host response?. Germs.

[37] Cooke C.L.M., Davidge S.T. (2003). Endothelial-dependent vasodilation is reduced in mesenteric arteries from superoxide dismutase knockout mice. Cardiovasc. Res..

[38] Gericke A., Sniatecki J.J., Mayer V.G., Goloborodko E., Patzak A., Wess J., Pfeiffer N. (2011). Role of M1, M3, and M5 muscarinic acetylcholine receptors in cholinergic dilation of small arteries studied with gene-targeted mice. Am. J. Physiol. Heart Circ. Physiol..

[39] McConnell K.W., Coopersmith C.M. (2016). Pathophysiology of septic shock: From bench to bedside. Presse Med..

[40] Ives S.J., Andtbacka R.H., Noyes R.D., Morgan R.G., Gifford J.R., Park S.Y., Symons J.D., Richardson R.S. (2013). alpha1-Adrenergic responsiveness in human skeletal muscle feed arteries: The impact of reducing extracellular pH. Exp. Physiol..

[41] Kuo J.H., Chen S.J., Shih C.C., Lue W.M., Wu C.C. (2009). Abnormal activation of potassium channels in aortic smooth muscle of rats with peritonitis-induced septic shock. Shock.

[42] Pedoto A., Nandi J., Oler A., Camporesi E.M., Hakim T.S., Levine R.A. (2001). Role of nitric oxide in acidosis-induced intestinal injury in anesthetized rats. J. Lab. Clin. Med..

[43] Fernandes D., Assreuy J. (2008). Nitric oxide and vascular reactivity in sepsis. Shock.

[44] Pi X., Xie L., Patterson C. (2018). Emerging Roles of Vascular Endothelium in Metabolic Homeostasis. Circ. Res..

[45] Durasevic S., Ruzicic A., Lakic I., Tosti T., Durovic S., Glumac S., Pavlovic S., Borkovic-Mitic S., Grigorov I., Stankovic S. (2021). The Effects of a Meldonium Pre-Treatment on the Course of the Faecal-Induced Sepsis in Rats. Int. J. Mol. Sci..

[46] Evgenov O.V., Pacher P., Schmidt P.M., Hasko G., Schmidt H.H., Stasch J.P. (2006). NO-independent stimulators and activators of soluble guanylate cyclase: Discovery and therapeutic potential. Nat. Rev. Drug Discov..

[47] Janssen B.J., De Celle T., Debets J.J., Brouns A.E., Callahan M.F., Smith T.L. (2004). Effects of anesthetics on systemic hemodynamics in mice. Am. J. Physiol. Heart Circ. Physiol..

[48] Constantinides C., Mean R., Janssen B.J. (2011). Effects of isoflurane anesthesia on the cardiovascular function of the C57BL/6 mouse. ILAR J..

[49] Cruz F.F., Rocco P.R., Pelosi P. (2017). Anti-inflammatory properties of anesthetic agents. Crit. Care.

