# Supplementary material for: Characterization of Systemic and Regional Hemodynamics and Vascular Dysfunction in Mice with Fecal Induced Peritonitis
Source: Biomedicines. 2022 Feb 18;10(2):470. doi: 10.3390/biomedicines10020470 (PMC8962278; doi:10.3390/biomedicines10020470)
Supplement: Supplementary file 1 [file biomedicines-10-00470-s001.zip › biomedicines-1571976-supplementary.pdf]

Supplementary Material

Characterization of Systemic and Regional Hemodynamics and Vascular Dysfunction in Mice with Fecal Induced Peritonitis

Forough Jahandideh [1,2], Sareh Panahi [1], Ronan M. N. Noble [2,3], Ferrante S. Gragasin [1,2], Rachel G. Khadaroo [4,5], Kimberly F. Macala [1,2,4] and Stephane L. Bourque [1,2,3,6,*]

1 Department of Anesthesiology & Pain Medicine, University of Alberta, Edmonton, AB T6G 2G3, Canada; jahandid@ualberta.ca (F.J.); sareh@ualberta.ca (S.P.); gragasin@ualberta.ca (F.S.G.); kmacala@ualberta.ca (K.F.M.)
2 Women and Children's Health Research Institute, University of Alberta, Edmonton, AB T6G 1C9, Canada; noble1@ualberta.ca
3 Department of Pediatrics, University of Alberta, Edmonton, AB T6G 2G3, Canada
4 Department of Critical Care Medicine, University of Alberta, Edmonton, AB T6G 2G3, Canada; khadaroo@ualberta.ca
5 Department of Surgery, University of Alberta, Edmonton, AB T6G 2G3, Canada
6 Department of Pharmacology, University of Alberta, Edmonton, AB T6G 2G3, Canada
* Correspondence: sbourque@ualberta.ca; Tel.: +1-780-492-6000

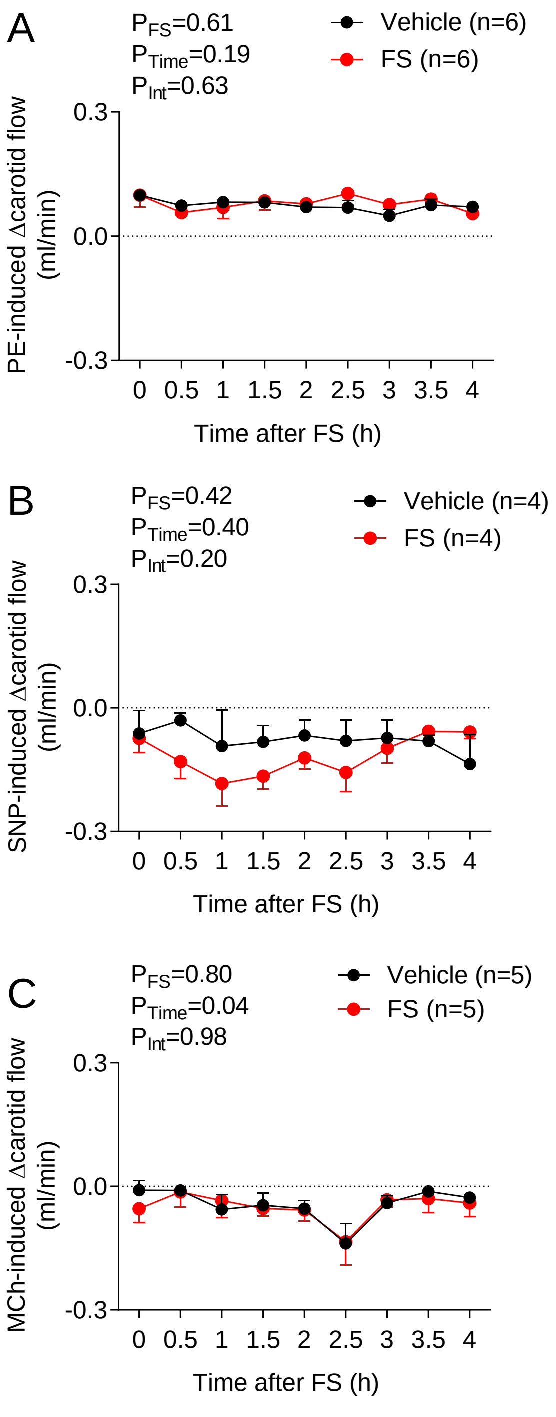

Figure. S1. Fecal-slurry-induced peritonitis (FIP) has little effect on carotid blood flow responses to repeat administration of bolus doses of (A) PE, (B) SNP, and (C) MCh in mice. N=6-7 mice per group. Data was analyzed by two-way analysis of variance (ANOVA) for effects of FS and time.

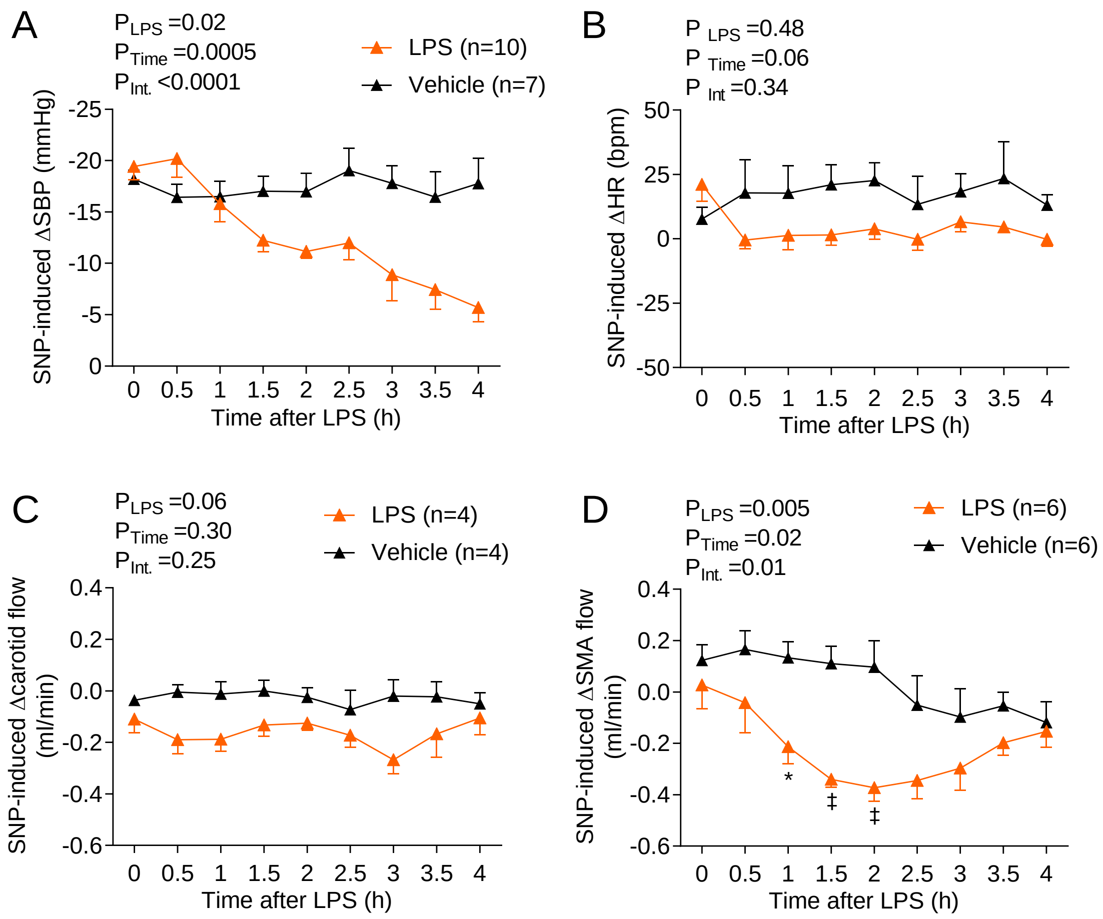

Figure. S2. Lipopolysaccharide (LPS) administration causes progressive reduction in systolic blood pressure response (A), no changes in heart rate (B) and carotid flow (C), and substantial reduction in SMA flow response to bolus doses of SNP (D) in mice. N=4-6 mice per group. Data was analyzed by two-way analysis of variance (ANOVA) for effects of FS and time, followed with Holm-Sidak post hoc test for multiple comparisons. *$P<0.05$ and ‡$P<0.001$ compared to vehicle at the same time.

Table S1. Biochemical parameters of blood gas analysis in fecal slurry (FS)-injected and vehicle groups

	**Vehicle**				**FS**				**P value**		
	**0.5-hour**	**1-hour**	**2-hour**	**4-hour**	**0.5-hour**	**1-hour**	**2-hour**	**4-hour**	**FS**	**Time**	**Int.**
**pCO_2 (mmHg)**	25.5±1.8	25.5±3.0	31.8±3.8	30.9±3.4	23.8±1.9	29.9±3.2	38.1±6.1	35.8±3.5	0.20	**0.03**	0.76
**pO_2 (mmHg)**	265.0±35.1	250.0±41.9	262.5±30.5	283.0±36.7	215.1±37.6	186.3±24.8	245.9±25.9	144.5±37.8*	**0.008**	0.58	0.32
**sO_2%**	99.95±0.03	99.95±0.03	99.75±0.10	99.90±0.00	99.08±0.46	98.96±0.54	99.82±0.02	99.55±0.20	**0.01**	0.47	0.21
**ctO_2 vol%**	19.6±0.5	19.4±0.7	19.4±0.8	18.7±0.4	22.3±0.8#	21.8±0.5#	20.9±0.9	21.0±1.2	**0.0002**	0.47	0.89
**Hct%**	42.98±8.64	41.71±1.39	41.25±1.28	40.14±1.01	48.17±1.38	47.29±1.02	44.86±1.92	48.75±2.17	**0.02**	0.86	0.85
**cHb (g/dL)**	13.46±0.45	13.53±0.48	13.41±0.39	13.06±0.33	15.77±0.45*	15.49±0.35*	14.66±0.63	15.88±0.71‡	**0.0002**	0.53	0.33
**cNa^+ (mmol/L)**	147.4±1.12	148.0±2.2	151.1±1.8	150.8±0.8	142.2±1.5	144.0±3.0	144.4±1.6*	150.0±1.7	**0.02**	**0.01**	0.26
**cK^+ (mmol/L)**	4.72±0.4	4.1±0.2	4.4±0.3	4.2±0.1	4.8±0.2	4.9±0.2	4.6±0.3	5.5±0.1†	**0.003**	0.42	0.07
**cCa^{2+} (mmol/L)**	1.4±0.0	1.3±0.0	1.4±0.0	1.4±0.0	1.4±0.0	1.4±0.1	1.4±0.0	1.4±0.0	0.78	0.70	0.50
**cCl^- (mmol/L)**	110.8±3.1	111.8±0.8	109.6±1.8	113.4±2.2	106.5±2.3	99.4±4.0	106.2±1.7	106.2±2.6	**0.03**	0.07	0.44
**cBase (mmol/L)**	-6.6±0.5	-8.8±1.4	-6.3±0.6	-7.2±0.9	-8.4±0.6	-8.2±0.9	-9.9±0.9*	-13.4±1.9‡	**0.001**	0.08	**0.02**
**$cHCO_3^-$ (mmol/L)**	20.1±0.4	19.2±0.7	18.8±0.7	19.0±0.6	19.0±0.3	18.5±0.8	16.5±1.0	14.0±1.4‡	**0.002**	**0.007**	**0.06**
**Lactate (mmol/L)**	3.3±0.4	3.3±0.4	4.4±0.4	4.0±0.4	5.1±0.5#	5.8±0.7*	6.2±0.6#	7.2±0.7‡	**<0.0001**	**0.06**	0.51
**pH**	7.44±0.02	7.44±0.04	7.36±0.03	7.38±0.03	7.42±0.02	7.36±0.04	7.27±0.06	7.21±0.04†	**0.002**	**0.003**	0.28

Data is presented as mean±SEM. N=5 mice per group. Data was analyzed by two-way ANOVA for effects of FS and time, followed with Holm-Sidak post hoc test for multiple comparisons. *P<0.05, †P<0.01, ‡P<0.001, # indicates P=0.06 compared to vehicle treated mice at the same time-point. sO_2: Oxygen saturation of hemoglobin, pO_2: Partial pressure of O_2, pCO_2: Partial pressure of CO_2, HCO_3^-: Standard bicarbonate, cBase (Ecf): Standard base excAess, ctO_2: Oxygen content.
